# Nucleobindin Co-Localizes and Associates with Cyclooxygenase (COX)-2 in Human Neutrophils

**DOI:** 10.1371/journal.pone.0002229

**Published:** 2008-05-21

**Authors:** Patrick Leclerc, Jordane Biarc, Mireille St-Onge, Caroline Gilbert, Andrée-Anne Dussault, Cynthia Laflamme, Marc Pouliot

**Affiliations:** Centre de Recherche en Rhumatologie et Immunologie and Department of Anatomy-Physiology, Faculty of Medicine, Laval University, Quebec City, Quebec, Canada; University of Arkansas, United States of America

## Abstract

The inducible cyclooxygenase isoform (COX-2) is associated with inflammation, tumorigenesis, as well as with physiological events. Despite efforts deployed in order to understand the biology of this multi-faceted enzyme, much remains to be understood. Nucleobindin (Nuc), a ubiquitous Ca^2+^-binding protein, possesses a putative COX-binding domain. In this study, we investigated its expression and subcellular localization in human neutrophils, its affinity for COX-2 as well as its possible impact on PGE_2_ biosynthesis. Complementary subcellular localization approaches including nitrogen cavitation coupled to Percoll fractionation, immunofluorescence, confocal and electron microscopy collectively placed Nuc, COX-2, and all of the main enzymes involved in prostanoid synthesis, in the Golgi apparatus and endoplasmic reticulum of human neutrophils. Immunoprecipitation experiments indicated a high affinity between Nuc and COX-2. Addition of human recombinant (hr) Nuc to purified hrCOX-2 dose-dependently caused an increase in PGE_2_ biosynthesis in response to arachidonic acid. Co-incubation of Nuc with COX-2-expressing neutrophil lysates also increased their capacity to produce PGE_2_. Moreover, neutrophil transfection with hrNuc specifically enhanced PGE_2_ biosynthesis. Together, these results identify a COX-2-associated protein which may have an impact in prostanoid biosynthesis.

## Introduction

Cyclooxygenase (COX) mediates a critical metabolic step in prostanoid biosynthesis. The inducible isoform, COX-2, largely predominates over the constitutive isoform, COX-1, in the production of prostaglandin (PG)E_2_ and thromboxane (TX)A_2_ in inflammatory cells including neutrophils [1]–[4]. PGE_2_ and TXA_2_ biosynthesis in neutrophils is initiated by the release of esterified arachidonic acid (AA) by type IV cytosolic (c)PLA_2_ [5]–[8]. COX-2 then catalyzes two reactions by which AA is transformed into PGH_2_, the common precursor to all prostanoids. PGH_2_ can be isomerized in PGE_2_, either non-enzymatically [9], or by the microsomal PGE_2_ synthase (mPGES)-1 isoform [8], while formation of TXA_2_ chiefly results from TXA_2_ synthase activity. It is well-established that PGE_2_ is implicated in pain, vasodilation and eodema. On the other hand, PGE_2_ inhibits major inflammatory responses of phagocytes. In neutrophils, PGE_2_ can prevent chemotaxis, aggregation, superoxide production, lysosomal enzyme release and generation of leukotriene B_4_ [4], [10]–[14].

The COX-2 enzyme has generated particular interest for its implication in inflammation, cellular proliferation, differentiation and tumorigenesis, [15], [16], and has recently emerged as a therapeutic target in the treatment and prevention of human cancers [17]–[20]. Also, COX-2 mediates physiological events such as kidney functions, post-natal development and female reproductive processes [21]–[23]. In spite of the pivotal roles of COX-2 in many aspects of biology, much remains to be discovered around the regulation of its activity in inflammatory cells. In particular, proteins that associate with COX-2 have yet to be identified.

Nucleobindin (Nuc) is a ubiquitous protein featuring multiple putative functional domains, indicating its potential implication in a number of cellular processes [24]–[28]. As such, Nuc has been the focus of reports originating from diverse fields including autoimmunity [29], intracellular signaling [30], osteogenesis [26], cancer [31] and inflammation [27]. At the protein level, Nuc is constituted of 460 amino acids, including an N-terminal 25 amino acid signal peptide responsible for its initial localization to the endoplasmic reticulum (ER) [32]. In addition, Nuc contains several classical interaction domains: a DNA binding site, a heterodimerization domain, two EF-hand Ca^2+^-binding sites, a nuclear localization signal [24]–[26] as well as non-classical protein-protein interaction domains including a G-protein-binding region and an high affinity COX-binding domain, as evidenced by a yeast two-hybrid assay [27], [28]. Depending on the model at hand, Nuc has been detected in various subcellular structures such as the nucleus [31], [33], mitochondria [34] the cytoplasm [34]–[37], the endoplasmic reticulum (ER) [33], [34], [36] and the Golgi apparatus [35]. The Golgi, like the ER, plays a role as an intracellular Ca^2+^ reservoir, which can be released in the cytosol in response to various stimuli, in turn activating a number of intracellular signaling cascades [38]. As such, Nuc may be involved in establishment of the agonist-mobilizable Golgi Ca^2+^ store [30]. However, notwithstanding a putative COX-binding site [27] and a relatively well-characterized capacity to bind Ca^2+^ [36], the biological functions of Nuc remain elusive.

In the present study, we investigated the expression of Nuc, its subcellular localization, its expression and affinity for COX-2, as well as its impact on COX-2-dependent PGE_2_ biosynthesis in human neutrophils. Results obtained identify Nuc as a COX-2-associated protein which may have a role in the biosynthesis of prostanoids.

## Methods

### Experimental Procedures

#### Materials

LR White was obtained from Reading (England). Polyclonal anti-albumin goat antibody was from CN Biosciences (La Jolla, CA, USA). Polyclonal anti-lactoferrin antibody was from Sigma (Oakville, ON, Canada). Monoclonal anti-GRP-78 was from BD Biosciences (Franklin Lakes, NJ, USA). Polyclonal anti-58k Golgi antibody was from Abcam Inc. (Cambridge, MA, USA). Polyclonal anti TXA_2_-synthase and monoclonal anti-COX-2 antibodies were from Cayman Chemical (Ann Arbor, MI, USA). Polyclonal anti-COX-2 antibodies were from BIOMOL International, L.P., (Plymouth meeting, PA, USA).

#### Cloning and purification

Full-length human Nuc was amplified using Expand HIFI+ DNA polymerase (Roche, Laval, Qc, Canada) from pOTB7/Nucleobindin clone (ID 2821805 produced by Invitrogen Life Technologies, Carlsbad, CA, USA). PCR reactions (35 cycles; annealing temp. 60°C) were performed using the following primers: 5′-GGA ATT TCA TAT GCC TCC CTC TGG-3′ (forward) and 5′-CCT AGC TCA TAT GTC ACA GAT GCT GG-3′ (reverse); yielding a PCR product of 1409 bp in length. pET/NucΔCBD was amplified from pET/Nuc using same DNA polymerase. PCR reaction (30 cycles; annealing temp. 60°C) was performed using the following primers: 5′-GGA ATT ACA TAT GAG TCC CGA CAC AGG-3′ (forward) and 5′-CCT AGC TCA TAT GTC ACA GAT GCT GG-3′ (reverse); yielding a PCR product of 1283 bp in length. Sequences of amplified fragments were confirmed by DNA sequencing. The cDNA products were cloned into the *Nde*I restriction site of pET-15b (Novagen, San Diego, CA, USA). BL21 cells were transformed with pET-15b/Nuc or pET/NucΔCBD expression vectors and induced with 1 mM isopropyl-1-thio-β-D-galactopyranoside (Tekniscience, Terrebonne, Qc, Canada). Bacterial extracts were processed for protein purification using a His-Bind Resin column and buffer kit (Novagen).

#### Production of polyclonal antibodies against nucleobindin

Rabbits were injected with an emulsion of 50–100 µg hrNuc and complete Freund's adjuvant. Total IgGs were purified using a Protein G-coupled sepharose® 4 fast flow column (GE Healthcare, Waukesha, WI, USA).

#### Human leukocyte isolation

Neutrophils were isolated as originally described [39] with modifications [4]. Viability was greater than 98%, as determined by trypan blue dye exclusion.

#### Cell incubations

Neutrophils were resuspended at a concentration of 5×10^6^ cells/ml (25×10^6^ cells/ml in experiments where RNA was to be extracted) in Hank's balanced salt solution (HBSS; 37°C) containing 10 mM HEPES pH 7.4, 1.6 mM Ca^2+^, no Mg^2+^ and the following antiprotease cocktail: 0.2 mg/ml diisopropylfluorophosphate (Serva Electrophoresis, Heidelberg, Germany), 10 µg/ml leupeptin, 10 µg/ml aprotinin (ICN Biomedicals Inc., Irvin, CA, USA).

#### PGE_2_ synthesis

In purified enzyme-based assays, 1.0 U hrCOX-2/sample were used (Cayman Chemicals) in HBSS 1× supplemented with Heme, in a final volume of 200 µl. Where indicated, hrNuc was added and samples were incubated for 15 min at 37°C. AA (10 µM final) was added and samples were incubated for 30 min at 37°C. Reactions were stopped by placing samples on ice-cold water. Samples were assayed for their content in PGE_2_ by ELISA (Assay Designs, Brockville, ON, Canada). Cross-reactivities in the PGE_2_ ELISA were <0.04% for 6-keto PGF_1α_, and <0.01% for LTB_4_, TXB_2_, and AA.

In cell-based assays, neutrophils stimulated with GM/TNF for 2 h were pelleted and resuspended in 700 µl ice-cold HBSS containing the anti-protease cocktail. Suspensions were sonicated on ice and centrifuged (3000× g). Cellular extract aliquots (5 µl) were incubated with hrNuc in a total of 200 µl for 15 min at 37°C, before stimulation with AA (10 µM final) for 30 min at 37°C. Reactions were stopped on ice-cold water; samples were briefly centrifuged and supernatants were assayed for their contents in PGE_2_ by ELISA.

#### Nitrogen cavitation

The procedure was conducted essentially as described [40], with modifications. For each cell preparation, 18 fractions were generated (1 ml each), starting from the bottom of the tube. This procedure allows the distinct separation of azurophil granules, specific granules, gelatinase granules, secretory vesicles, a plasma membrane-enriched fraction, and cytosol [40]. Each fraction was re-centrifuged (100 000× *g*, 90 min) in a Beckman TL 100 ultracentrifuge, using a TL 100.2 rotor, in order to pellet Percoll. Fractions (50 µl) were carefully aspirated with a pipet and processed for western immunoblot analysis.

#### Western immunoblots

Samples, resuspended in sample buffer 1× (62 mM Tris-HCl, pH 6.8, 2% SDS, 2.5% β-mercaptoethanol, 10% glycerol, with antiprotease cocktail), were subjected to 10% SDS-PAGE and transferred to Immobilon membranes (Millipore Corp., Bedford, MA, USA). The membranes were soaked for 30 min at RT in Tris-buffered saline (TBS: 25 mM Tris-HCl pH 7.6, 0.2 M NaCl, 0.15% Tween 20) containing 5% (w/v) dried milk, and exposed for 60 min with the first antibody. Membranes were then washed twice in TBS, and incubated for 30 min with a 1∶10 000 dilution of a horseradish peroxidase (HRP)-linked donkey anti-rabbit antibody (Biocan Scientific, Mississauga, ON, Canada), or HRP-linked sheep anti-mouse antibody (GE Healthcare). Enzyme expression was revealed with ECL-Plus (Perkin Elmer, Boston, MA, USA).

#### Immunofluorescence

Neutrophils were fixed in 4% paraformaldehyde for 20 min, then washed twice with PBS. Cell suspensions were laid onto poly-L-lysine-coated glass slides and allowed to air-dry. Slides were incubated with a permeabilization buffer (0.5% NP-40, 5% heat-inactivated fetal bovine serum (FBS) and 5% heat-inactivated donkey serum in PBS) for 5 min, washed three times in washing buffer (5% FBS, 0.05% NP-40 in PBS), incubated with a blocking buffer (10% FBS and 10% donkey serum in PBS) for 30 min and washed three times prior to a 1 h incubation with an anti-Nuc antibody (diluted 1/150 in: PBS with 5% FBS, 5% donkey serum and 0.05% NP-40) in a humid environment. After washes, slides were incubated with goat anti-rabbit AlexaFluor® 488 (Molecular Probes, Carlsbad, CA, USA; diluted 1/200) for 30 min in the dark, in a humid environment. Slides were washed once and then incubated with DAPI 0.7 µmoles/ml (Molecular Probes) or with 250 ng/ml propidium Iodine (PI; Sigma) for 5 min in the dark. Slides were washed in PBS and prepared for microscopy with Gel/Mount™ (Biomeda, Foster City, CA, USA). Images were captured by a CoolSNAP HQ camera mounted on an Olympus BX-51 upright microscope using a 60× UPlan Apo objective, and processed with ImagePro 4.5.1 software (Media Cybernetics, Silver Spring, MD, USA). Confocal microscopy was performed on an Olympus BX-61 microscope using a UPlan Apo 100× objective with immersion oil. Data was collected with the FluoView software (Olympus).

*Movie S1:* The 3D representation was generated with confocal microscopy images, using ImageJ (http://rsb.info.nih.gov/ij) and edited with iMovie HD (Apple Computer Inc.).

#### Immunogold labeling and electron microscopy

Following stimulation with GM/TNF, cells were fixed for 24 h in a solution 0.1 M cacodylate buffer [Na(CH_3_)_2_AsO_2_⋅3H_2_O], pH 7.3, containing freshly prepared 4% paraformaldehyde and 0.2% glutaraldehyde. Fixed cells were then washed 3 times with 0.1 M cacodylate buffer pH 7.3 and dehydrated in ethyl alcohol by gradually increasing the specimen's ethyl alcohol concentration (30%, 50%, 70% and 90%, 10 min twice per concentration). The specimen were infiltrated with LR White (LR White/90% ethanol 1∶1 mixture) 2 h at RT, overnight at 4°C and 2 times 2 h at RT. LR White polymerization was triggered by subjecting the specimen to U.V. light for 48 h at 4°C. Embedded specimens were cut into ultrathin sections (80 nm), then laid on Ni/fomvar grids. Grids were blocked with PBS 1× + 5% BSA for 30 min, RT and washed with PBS 1× + 1% BSA + 0.1% Tween-20. Grids were deposited face down on a 50 µl drop of antibody solution. Incubations with primary antibodies (rabbit polyclonal anti-Nuc, 1/50; chicken polyclonal anti-Nuc, 1/100 000, Genway Biotech, San Diego, CA, USA); polyclonal anti-COX-2 (1/50, Santa Cruz Biotechnologies, Santa Cruz, CA, USA); polyclonal anti-GRP-94 (1/400, Abcam Inc.) were done overnight at 4°C, whereas incubations with secondary antibodies, either 18 nm colloidal Gold-AffiniPure® donkey anti-chicken IgY or 6 nm colloidal Gold-AffiniPure® goat anti-rabbit IgG (Jackson Immunoresearch Labs., West Grove, PA, USA), were done at RT for 4 h. The double labeling was performed in a serial fashion starting with the Nuc labeling followed by the COX-2 labeling. After each antibody incubation, grids were washed with 5×1 ml PBS 1× + 1% BSA + 0.1% tween20. Following the last antibody incubation, the grids were washed with 5×1 ml dH_2_O. Finally, the dried grids were stained with uranyl acetate. Analyses were performed on a JEOL JEM-1230 transmission electron microscope (JEOL, Montreal, QC, Canada). Magnifications were between 10 000× and 120 000×.

#### Immunoprecipitations with magnetic beads

Anti-Nuc antibodies generated in-house, or irrelevant IgGs, were coupled to Dynabeads M-500 subcellular (Dynal, Norway). Immunomagnetic beads were incubated with selected subcellular fractions at a ratio of 10 µl fraction/5 million beads/ml in PBS pH 7.4, 2 mM EDTA, 5% BSA and the anti-protease cocktail, for 12 h at 4°C. Beads were magnetically immobilized and supernatants were discarded. The beads were washed three times in PBS pH 7.4, 2 mM EDTA, anti-protease cocktail, with decreasing concentrations of BSA (5%, 0.1%, 0%), then resuspended in sample buffer 1× and boiled for 2–3 min.

#### Pull-down assay

hrNuc was coupled to CNBr-activated Sepharose® 4B beads (GE Healthcare). PMA-stimulated cells were pelleted (microfuge) and resuspended in 0.1% NP-40 lysis buffer [41] for 10 min at 4°C. The lysates were centrifuged at 1000× *g* for 10 min at 4°C. In this procedure, COX-2 is mainly located in the non-nucleus fraction, which was used for the present assay. Supernatants were incubated with 50 µl of hrNuc-coupled sepharose beads, or with inactivated sepharose beads for 2 h, RT. After incubation, beads were centrifuged (30 sec, 1500× *g*), washed twice with HBSS + CaCl_2_ + antiprotease cocktail, resuspended in sample buffer 1× and heated 2–3 min at 95°C.

#### Immunoprecipitations under native conditions

Immunoprecipitations were performed as described earlier [42], with modifications. Briefly, neutrophils stimulated with GM/TNF were centrifuged, and the cell pellets were lysed by adding cold lysis buffer (10 mM Tris-HCl, pH 7.4, 137.2 mM Nacl, 1 mM EDTA, 0.6% CHAPS, 2 mM orthovanadate, and the protease inhibitor cocktail) for 5 min on ice. The insoluble material was discarded after centrifugation at 13 000× g at 4 °C during 5 min. The supernatant was harvested, then precleared with protein A-Sepharose at 4 °C for 30 min. Resulting supernatants were incubated at 4 °C either with 8 µg of anti-COX2 (mouse) antibodies or 8 µg of anti-Nuc (rabbit) antibodies for 1 h followed by 2 h incubation with protein A-Sepharose beads. The beads were collected and washed three times with cold lysis buffer. Laemmli sample buffer (2×) was added to the beads, which were boiled for 7 min.

#### Transfection of recombinant nucleobindin

We used the Pro-Ject Protein Transfection Reagent (Pierce, Rockford, IL), according to the manufacturer's instructions. Briefly, neutrophils were stimulated with GM-CSF/TNF-α at 37°C. After 15 min, a mixture of 7.5 µl of the reagent with 2 µg of the protein (Nuc or NucΔCBD) were added and incubated for 4 h at 37°C. AA (10 µM) was added and samples were incubated for an additional 30 min at 37°C. Samples were centrifuged and supernatants were assayed for PGE_2_ content by ELISA. Cell pellets were processed for SDS-PAGE.

#### Statistical analysis

Where applicable, statistical analysis was performed by Student's non-paired t-test (two-tailed), and significance (*, **) was considered attained when *p* was <0.05.

## Results

### Nucleobindin is constitutively expressed in human phagocytes

**Figure 1:**
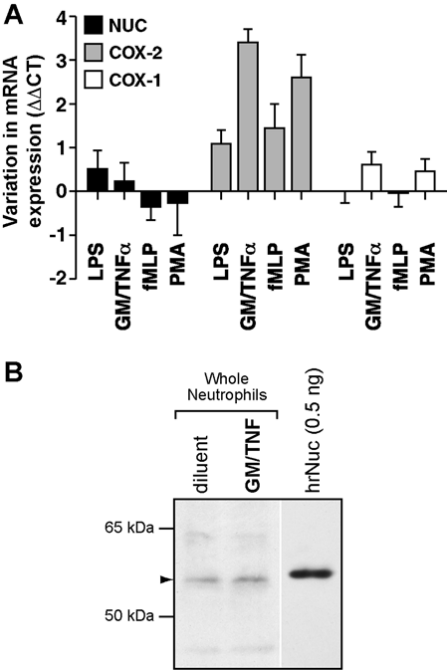
Expression of Nuc in human neutrophils. A) Real-time PCR determination of Nuc, COX-1 and COX-2 messenger RNA expression in neutrophils. Cells were stimulated for 60 min with lipopolysaccharide (LPS ; 100 ng/ml), a mixture of granulocyte/monocyte colony stimulation factor and tumor necrosis factor-α (GM/TNF; 1.4 nM and 100 ng/ml respectively), formyl-methionyl-leucyl-phenylalanine (fMLP; 100 nM) or with PMA (10 nM). Samples were processed for the determination of GAPDH, COX-1, COX-2 and Nuc mRNA expression by real-time-PCR. Shown are integrated results from n = 4 (±SEM) separate experiments performed in identical conditions with different donors. B) Nuc protein expression in neutrophils, as determined by western immunoblotting. Cells were incubated for 2 h with diluent (saline), or with GM/TNF. Samples were processed for the determination of Nuc expression by western immunblotting. Nuc is constitutively present in unstimulated neutrophils. Note that hrNuc migrated slightly slower than neutrophil native Nuc, due to the presence of the signal peptide and of the additional His-Tag sequence. Shown is one immunoblot, representative of four identical experiments performed with different donors.

Expression of Nuc in neutrophils was first assessed. To this end, cells were incubated with agonists known to stimulate inflammatory gene expression in these cells: lipopolysaccharide (LPS), the formylated synthetic peptide fMLP, the phorbol ester PMA, or a mixture of granulocyte-macrophage colony-stimulating factor and tumor necrosis factor-α (GM/TNF). Following stimulations, mRNA levels of COX-1, COX-2 and Nuc were determined by real-time PCR. While each of the agonists elicited an increase in expression of COX-2 mRNA, as previously reported [3], [43], that of Nuc only varied in a modest fashion and, in most conditions, variations were comparable to that of COX-1, a constitutively-expressed gene in neutrophils (Fig. 1A). Similar results were obtained in human monocytes stimulated with LPS (data not shown). These results are consistent with the reported structure for the promoter region of the Nuc gene, featuring typical elements of house-keeping genes [32]. Data obtained at the protein level also indicate that resting neutrophils constitutively express Nuc (Fig. 1B).

### Nuc and COX-2 are localized in the Golgi and ER of neutrophils

**Figure 2:**
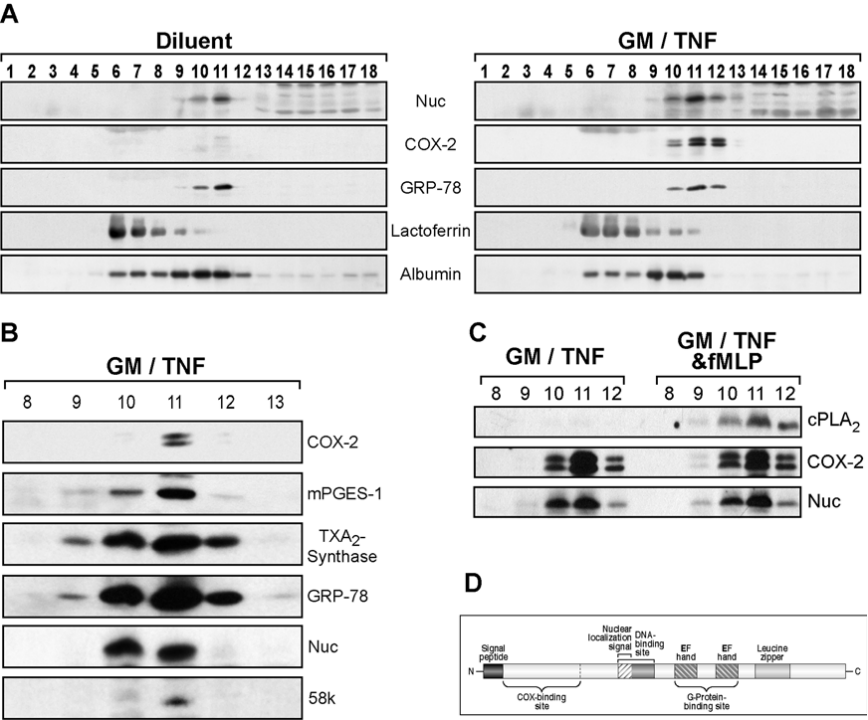
Neutrophil subcellular fractionation and localization of Nuc. A) Resting (left panel) or GM/TNF-stimulated (right panel) neutrophils were processed for cavitation and subcellular fractionation, as described in *Experimental procedures*. Nuc and COX-2 co-localized in ER/Golgi-containing fractions, as determined by western immunoblotting. B) In GM/TNF-stimulated neutrophils, samples were processed as in A) for the determination of the indicated proteins. C) In GM/TNF, and GM/TNF+fMLP (100 nM) stimulated neutrophils, samples were processed as in A) for the determination of the indicated proteins. In each panel, all immunoblots originate from the same membrane. Shown is one immunoblot, representative of four identical experiments performed with different donors. *GRP-78:* ER/Golgi marker; *lactoferrin:* marker of specific granules; *albumin*; marker of secretory vesicles; *58k:* 58k Golgi protein (Golgi marker); *mPGES-1:* microsomal prostaglandin E_2_ synthase-1; *TXA_2_-Synthase:* thromboxane A_2_ synthase; *cPLA_2_:* Type IV cytosolic phospholipase A_2_. D) Schematized protein structure of human Nuc and putative functional domains. The main characterized domains found in Nuc are: a signal peptide directing the protein to the ER; a COX-binding site; a putative nuclear localization signal embedded in to a DNA-binding site; two EF-hand Ca2+-bindins sites; a leucine zipper region.

The subcellular localization of Nuc, and of the enzymatic machinery responsible for prostaglandin biosynthesis was investigated in human neutrophils. To this end, we used the nitrogen cavitation technique coupled to fractionation on a Percoll® density gradient, a well-recognized procedure which allows for the separation of intracellular compartments such as: four distinct populations of granules, secretory vesicles, plasma membranes and the cytosol [44]. Neutrophils were stimulated for 2 h with GM/TNF, a condition which efficiently up-regulates their expression of COX-2 [3]. Following stimulation, cells were processed for nitrogen cavitation, subcellular fractionation, and detection of proteins of interest by western immunoblotting. The fractionation pattern was validated with the use of specific cellular compartment markers. Lactoferrin, a marker of specific granules, was predominantly found in fractions 6 to 8, whereas albumin, the marker for secretory vesicles, was predominantly in fractions 9 to 11 (Fig. 2A), in accordance with previous reports [44], [45]. Nuc was mainly found in fraction 11, both in diluent- and in GM/TNF-stimulated cells. When COX-2 was up-regulated, it co-localized with Nuc, in the fractions 10 to 12 of GM/TNF-stimulated cells (Fig. 2A, right panel) and also with the glucose-related protein (GRP)-78, marker for Golgi and ER structures. Localization patterns of Nuc, COX-2 and GRP-78 systematically matched with each other, suggesting that Nuc and COX-2 both reside in the Golgi and ER structures [44].

Additional fractionation experiments were performed with GM/TNF-stimulated neutrophils in order to localize additional enzymes implicated in prostanoid synthesis. Microsomal PGE_2_-synthase-1, thromboxane-synthase, as well as 58k, an additional marker for the Golgi, also co-localized with COX-2 and Nuc-positive fractions (Fig. 2B). For the specific case of type IV cytosolic phospholipase A_2_ (cPLA_2_), GM/TNF-treated cells were stimulated with fMLP prior to the fractionation process in order to induce its phosphorylation and translocation to membranes [41]. In this situation, cPLA_2_ was readily detected in COX-2- and Nuc-containing fractions (Fig. 2C).

### Nuc and COX-2 are localized in proximity of each other in neutrophils

**Figure 3:**
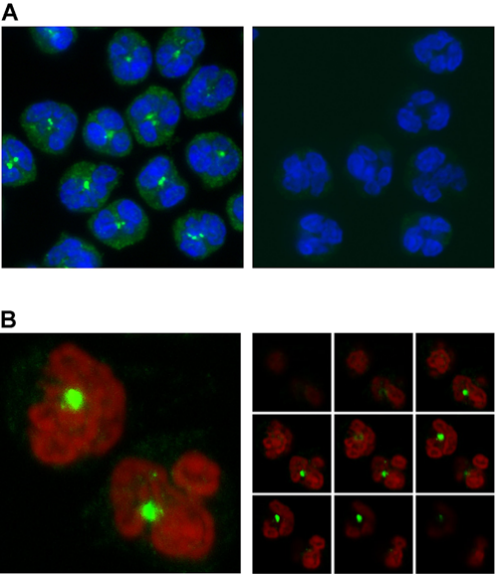
Subcellular localization of Nuc in neutrophils, as assessed by indirect immunofluorescence and confocal microscopy. Resting neutrophils were processed for the detection of Nuc by immunofluorescence, as described in *Experimental Procedures*. A) Nuclei were stained in blue. Immunoreactive Nuc, in green, was mainly observed in the center of cells, typically as two to three main spots between nuclear lobes and, to a lesser extent, within the cytoplasm. *Left panel:* with anti-Nuc antibody. *Right panel:* anti-Nuc antibody was omitted. B) For confocal microscopy experiments, the nucleus was stained with propidium iodine and appeared red-orange; darker regions are distinctive of euchromatin; immunoreactive Nuc, in green, appeared at the center of the cell. *Left panel:* composite confocal image; most of the immunoreactive Nuc was at the center of the cell, with a limited number of smaller spots also in the vicinity of the nucleus. *Right panel, (from left to right, top to bottom):* represented are 9 equidistant slices from the composite image shown in left panel.

**Figure 4:**
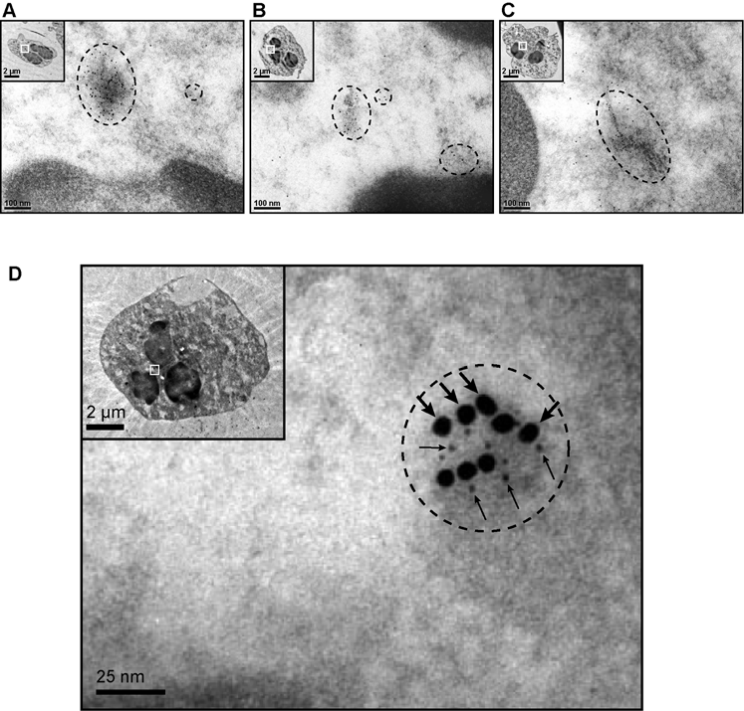
Demonstration of co-localization for Nuc and COX-2 in neutrophils, by electron microscopy. Neutrophils stimulated with GM/TNF were processed for the detection of Nuc (A), COX-2 (B) and Golgi (C), by indirect immunostaining and electron microscopy. For each protein, labeling was mainly found in a single cluster situated between nuclear lobes, in the center of the cell. D) Samples were processed for the double detection of Nuc and of COX-2 by electron microscopy. To this end, a polyclonal chicken anti-Nuc antibody and a polyclonal rabbit anti-COX-2 antibody, were used in sequence, as described in the *Experimental procedures*. Co-localization of Nuc (18 nm beads, indicated by thick arrows) and COX-2 (6 nm beads, thin arrows) is clearly seen in a cluster between nuclear lobes. In each panel, a single neutrophil is shown in the upper-left corner; the magnified region of interest is represented by the respective white square.

Results obtained so far suggest a Golgi and ER localization for Nuc and for the enzymatic machinery mediating prostanoid synthesis. We sought further confirmation for this co-localization in intact cells, by immunofluorescence, confocal micrscopy, and electron microscopy. For immunofluorescence experiments, resting neutrophils were fixed and permeabilized, then processed for the detection of Nuc by indirect labeling. As can be appreciated in Fig. 3A (left panel), immunoreactive Nuc (green labeling) appeared predominantly embedded near the center of the cells and between nuclear lobes, typically with one or two main spots per cell and a small number of secondary spots, a pattern consistent with a Golgi and ER localization [46]. Analysis of the samples by confocal microscopy further precised the central location of Nuc within the cell (Fig. 3B). A 3D representation of the confocal data (*Movie S1*) nicely illustrates the central localization of Nuc within the neutrophil, consistent with its Golgi and ER localization. In electron microscopy experiments, intact neutrophils stimulated with GM/TNF were fixed and embedded; ultrathin slices were incubated with specific polyclonal antibodies for the detection of Nuc (Fig. 4A), COX-2 (Fig. 4B) or the Golgi marker GRP-94 (Fig. 4C). For each of the three proteins, labeling was found mainly clustered in a central area localized in the vicinity of nuclear lobes, and in a small number of secondary sites, largely confirming the immunofluorescence and confocal microscopy data and supporting the idea that Nuc and COX-2 both localize in the Golgi and ER. In a separate set of experiments, samples were subjected to a double-labeling and prepared for electron microscopy showed proximity between COX-2 and Nuc (Fig. 4D). The pattern of labeling demonstrated that both proteins can be located very close to each other, clustered on the luminal side of vesicular structures (Nuc, thick arrows; COX-2, thin arrows). Experiments performed with resting neutrophils yielded virtually no COX-2 labeling (data not shown).

### Nuc binds to COX-2 with high affinity

**Figure 5:**
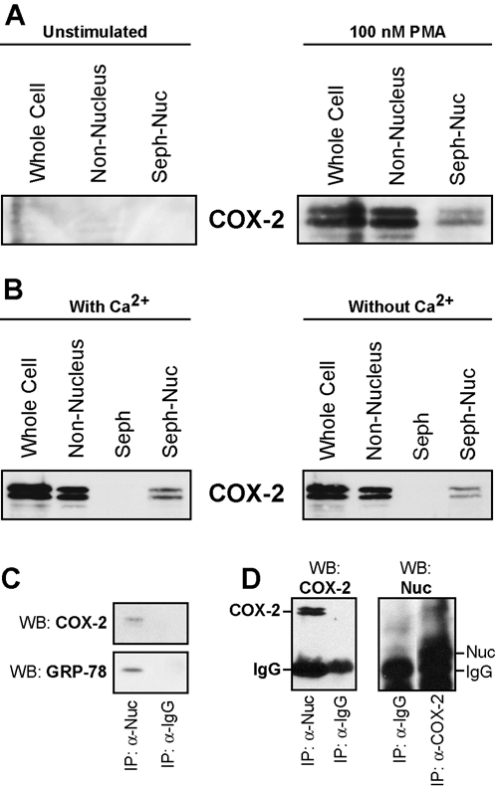
Direct interaction between neutrophil Nuc and COX-2. A) Nuclear-free extracts from unstimulated or PMA-stimulated neutrophils were incubated with sepharose beads linked to hrNuc for a pull-down assay, then processed for western immunoblotting for the detection of COX-2. Lane 1: whole cells, lane 2: nucleus-free extracts, lane 3: nucleus-free extracts incubated with sepharose beads linked to hrNuc (Seph-Nuc). B) Nuclear-free extracts from PMA-stimulated cells were processed as in A), in presence of 1.5 mM Ca^2+^ (left panel) or in presence of 5 mM EGTA (right panel; without Ca^2+^). Lane 1: whole cells, lane 2: nucleus-free extracts, lane 3: nucleus-free extracts incubated with inactivated sepharose beads, lane 4: nucleus-free extracts incubated with sepharose beads linked to hrNuc (Seph-Nuc). For each panel, results shown are from one experiment, typical of two separate experiments performed in identical conditions with different donors. C) Immunomagnetic beads coated with anti-Nuc IgGs or with irrelevant IgGs were incubated with an aliquot from the positive fractions (10 to 12) showed in Fig. 1. Anti-Nuc-coated beads immunoprecipitated a structure that was positive for COX-2 and GRP-78. Results are from one experiment, representative of n = 3 distinct experiments performed in identical conditions. D) *Left panel*: Nuc was immunoprecipitated from COX-2-expressing neutrophils, using anti-Nuc or irrelevant anti-IgG antibodies, as described in the *Experimental procedures*; samples were processed for the detection of COX-2 by western immunoblot. *Right panel*: COX-2 was immunoprecipitated using anti-COX-2 or irrelevant anti-IgG antibodies and samples were processed for the detection of Nuc by western immunoblot (IP: Immunoprecipitation; WB: western immunoblot). Results are from one experiment, representative of n = 2 distinct experiments performed in identical conditions.

Affinity between Nuc and COX-2 [27] was confirmed in neutrophils by incubating sepharose beads coated with hrNuc with lysates obtained from resting- or PMA-stimulated neutrophils. COX-2 co-immunoprecipitated along with hrNuc, assessed by western immunoblottings (Fig. 5A). Chelation of Ca^2+^ with 5 mM EGTA did not prevent immunoprecipitation of COX-2 by hrNuc (Fig. 5B), indicating that the association between the two proteins does not chiefly rely on availability of Ca^2+^. Presence of Nuc and COX-2 within the same organelle was also demonstrated by immunoprecipitation experiments. An aliquot of Nuc-positive fractions resulting from nitrogen cavitation experiments (right panel of Fig. 2A, fraction 11) was incubated with anti-Nuc polyclonal antibodies covalently-linked to magnetic beads. These anti-Nuc-coated beads specifically immunoprecipitated structures which, in addition to containing Nuc, also contained COX-2 and GRP-78 (Fig. 5C), showing that the organelles containing Nuc also harbor COX-2 and Golgi/ER structures. Finally, direct association between Nuc and COX-2 was demonstrated by two ways through immunoprecipitation experiments. First, COX-2-expressing neutrophil lysates were treated with an anti-Nuc antibody and presence of COX-2 in the immunoprecipitates was confirmed by western immunoblots (Fig. 5D, left panel). Second, lysates were conversely treated with an anti-COX-2 antibody, and Nuc could also detected in immunoprecipitates (Fig. 5D, right panel). Together, these results demonstrate co-localization, proximity and direct association between Nuc and COX-2 in human neutrophils.

### Nucleobindin increases cyclooxygenase-2-dependent prostaglandin E_2_ generation

**Figure 6:**
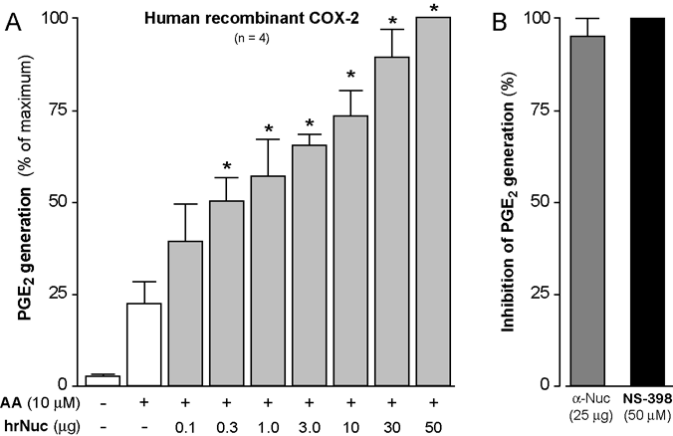
Nuc increases COX-2-dependent PGE_2_ biosynthesis. A) Recombinant human (hr)COX-2 (1 U/sample) alone or in presence of indicated amounts of hrNuc, were incubated with AA. PGE_2_ production was measured by ELISA. Results are expressed as percentages of maximum production and are the mean±s.e.m. of four separate experiments performed in identical conditions. B) hrNuc (10 µg) was treated with a polyclonal anti-Nuc antibody prior to incubation with hrCOX-2. The COX-2 specific inhibitor NS-398 (50 µM) was used to confirm a COX-2-mediated event. Results are expressed as percentage of inhibition of PGE_2_ production, when compared to the production obtained in the absence of antibody or inhibitor (mean±s.e.m., n = 4. *: significantly higher than samples incubated without hrNuc; **: significantly higher than samples incubated with 2.0 µg hrNuc or less).

We addressed the intriguing possibility that Nuc may impact on COX-2 activity, first by using an *in vitro* enzymatic assay with purified human recombinant (hr)COX-2. In this highly-simplified system, addition of hrNuc increased the generation of PGE_2_ in a concentration-dependent fashion, up to 4 fold over basal levels (Fig. 6A). Pre-treatment of Nuc with the anti-Nuc polyclonal antibody prevented the increase in PGE_2_ production (Fig. 6B), pointing to a specific implication of Nuc in this process. In addition, the Nuc-enhanced PGE_2_ production was entirely prevented by the specific COX-2 inhibitor NS-398, confirming a COX-2 mediated event (Fig. 6B). These results, showing enhancing impact of Nuc on COX-2-dependent PGE_2_ production, support the concept of a physiologically significant interaction between Nuc and COX-2.

**Figure 7:**
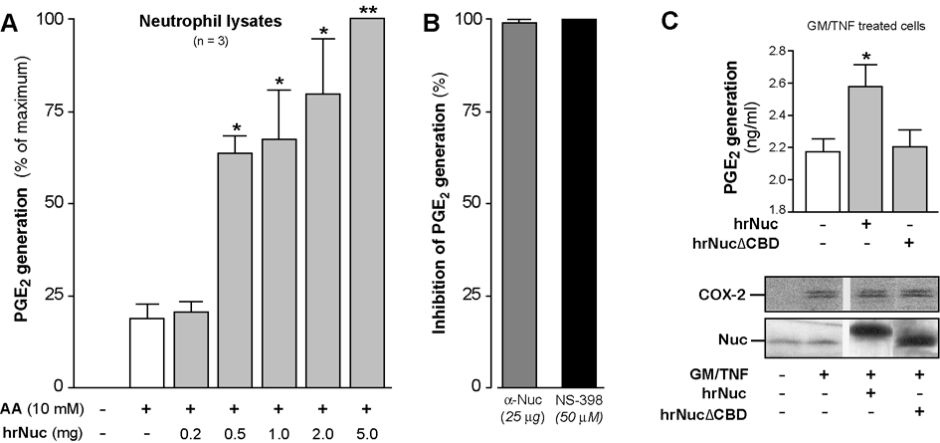
Nuc increases PGE_2_ biosynthesis in a COX-2-dependent manner in human neutrophils. A) COX-2-expressing neutrophil extracts were incubated with AA (10 µM) for 30 min, alone or in the presence of indicated quantities of hrNuc. PGE_2_ production was measured by ELISA. Results are expressed as percentages of maximum PGE_2_ production (mean±s.e.m., n = 3). B) Left bar; hrNuc (1 µg) was treated with a polyclonal anti-Nuc antibody prior to incubation with the cellular extracts. Right bar; the COX-2 specific inhibitor NS-398 (50 µM) was used to confirm a COX-2-mediated PGE_2_ biosynthesis. Results are expressed as inhibition of PGE_2_ production, when compared to the production obtained in the absence of antibody or inhibitor (mean %±s.e.m., n = 4). C) Bar graph; GM/TNF-treated neutrophils were transfected with full-length hrNuc, or lacking a COX-binding domain (NucΔCBD) using the Pro-Ject procedure as described in *Experimental procedures*, then stimulated with AA (10 µM). PGE_2_ production was measured by ELISA. (mean±s.e.m., n = 3. *: significantly higher than samples incubated without Nuc). Western immunoblots; Cells treated as described above were processed for the determination of cellular COX-2, Nuc, hrNuc and NucΔCBD protein levels. Note that hrNuc migrates higher than endogenous Nuc (or NucΔCBD), because of the signal peptide sequence still being present. Immunoblots are from one experiment, typical of three independent experiments performed in identical conditions.

This point was specifically addressed: lysates from COX-2-expressing neutrophils were stimulated with AA, alone or in the presence of increasing quantities of hrNuc, and production of PGE_2_ was measured. In these experiments, exogenous hrNuc increased the production of PGE_2_ in a concentration-dependent fashion, up to 5-fold over basal levels (Fig. 7A). And, once again, the Nuc-enhanced PGE_2_ production was prevented by pre-incubation of hrNuc with an anti-Nuc antibody, or by the presence of NS-398 (Fig. 7B), confirming a Nuc and COX-2-mediated event. Further evidence of a functionally relevant interaction between Nuc and COX-2 was obtained by transfecting Nuc into intact COX-2-expressing neutrophils. Cells were transfected either with full-length hrNuc or with hrNuc lacking the COX-binding domain (NucΔCBD), then stimulated with AA. As can be appreciated in Fig. 7C, addition of full length hrNuc specifically caused a significant increase in PGE_2_ biosynthesis by neutrophils, while NucΔCBD was ineffective.

## Discussion

All approaches taken, be that cavitation coupled to subcellular fractionation, electron microscopy, immunofluorescence, or confocal microscopy, indicated that Nuc mainly clusters in a central place in the vicinity of -but distinct from- nuclear lobes, a region in which the Golgi resides [46], and in a small number of nearby additional spots, consistent with ER localization. Conversely, Nuc does not appear to be present in granules or in the plasma membrane of human neutrophils. It remains possible that lower levels of Nuc could be found elsewhere, but the thorough approach undertaken herein strongly points to a Golgi/ER localization for Nuc.

In different cell types , COX-2 has also been reported to localize in the Golgi and ER. In addition to an N-terminal signal peptide which causes initial ER integration during translation, a C-terminal KDEL-like signal (PTEL) is also present in the structure of COX-2 and thought to be recognized by a membrane-bound receptor that continually retrieves the proteins from later compartments of the secretory pathway and returns them to the ER [47]. Results from the present study further document a Golgi and ER localization for Nuc and COX-2 in neutrophils. In addition, activated cPLA_2_, mPGES-1 and TXA_2_- synthase, in fact all enzymes of the prostanoid biosynthesis machinery, were also found in the same Golgi- and ER-containing fractions, along with Nuc and COX-2. Fractionation procedures and microscopy experiments further showed that, in human neutrophils, Nuc and COX-2 can localize in proximity of each other. Finally, immunoprecipitations and pull down assays each confirmed direct interaction and a high affinity between Nuc and COX-2.

When focusing on the delineation of a physiologically-relevant function for this high-affinity association, hrNuc specifically increased the COX-2-mediated formation of PGE_2_, in three distinct settings: purified COX-2, cell lysates, and transfected intact cells. Amongst all known prostanoids, purified human neutrophils only release PGE_2_ and TXA_2_ from COX-2 activity [3]. In turn, only PGE_2_ was considered in this study as it can be non-enzymatically produced from COX-generated PGH_2_. The increase in COX-2-dependent PGE_2_ generation was concentration-dependent and reached up to five fold increase, relative to basal levels. Also, presence of a putative COX-binding domain [27] was necessary for increasing PGE_2_ production. These experiments could not, however, take into consideration crucial factors such as the micro-environment, compartmentalization, or cellular architecture. Moreover, post-translational modifications of native Nuc, absent in hrNuc, may affect interactions in an as of yet unknown fashion. In this regard, Nuc does not appear to be *N*- or *O*-linked glycosylated, but possesses up to 10 potential phosphorylation sites including three protein kinase C sites which may well impact on its conformation and propensity to associate with other proteins, including COX-2 [26]. In turn, further studies documenting a thorough determination of the stoichiometry of this association, as well as of involvement of final-step enzymes (e.g., mPGES-1, TXA_2_-S), which will most likely require combinations of cell-free and cell line- based experimental set ups, will be necessary before the architecture of this pivotal enzymatic complex can be progressively unveiled. Nonetheless, results obtained indicate a high affinity between Nuc and COX-2.

In summary, we found that Nuc mainly localizes in the Golgi and ER of human neutrophils, along with COX-2 and other enzymes involved in prostanoid generation. Nuc can associate with COX-2 with high affinity and increase the resulting PGE_2_ generation. The potential role of Nuc in the regulation of PGE_2_ production is of interest in a large number of physiological settings, and the present report might be a first step in a characterization of this pivotal enzymatic complex.

## Supporting Information

Movie S1 3D visualization of Nuc localization. A 45 sec video sequence, based on confocal data obtained on the sample presented in B), illustrates the 3 dimensional localization of Nuc in the center of one human neutrophil. Nuc is clearly observed in the center of the cell and within a limited number of vesicles in the vicinity of the nucleus, typical of the Golgi and endoplasmic reticulum, respectively. The second half of the video is in ‘edge’ mode, which delineates the contours of localization of Nuc, putting in evidence a central reservoir at the center of the cell as well as close-by endoplasmic reticulum vesicles.
(3.36 MB MOV)
